# Decreased risk of radiation pneumonitis with concurrent use of renin-angiotensin system inhibitors in thoracic radiation therapy of lung cancer

**DOI:** 10.3389/fmed.2023.1255786

**Published:** 2023-10-12

**Authors:** Yawen Zheng, Changsheng Cong, Zewen Wang, Yanan Liu, Mingyan Zhang, Hao Zhou, Chen Su, Meili Sun

**Affiliations:** ^1^Department of Oncology, Central Hospital Affiliated To Shandong First Medical University, Jinan, China; ^2^Department of Oncology, Jinan Central Hospital, Shandong University, Jinan, China

**Keywords:** radiation pneumonitis, renin-angiotensin system inhibitors, lung cancer, thoracic radiation, predictive factors

## Abstract

**Background:**

Radiation pneumonitis (RP) is the primary dose-limiting toxicity associated with radiotherapy. This study aimed to observe the effects of renin-angiotensin system inhibitors in Chinese patients with lung cancer who received thoracic radiation.

**Methods:**

Patients with lung cancer who received thoracic radiation at a total dose of ≥45 Gray between October 2017 and December 2022 were enrolled in this study. We retrospectively evaluated the factors influencing grade 2 or higher RP.

**Results:**

A total of 320 patients were enrolled in this study; 62 patients were identified as angiotensin receptor blockers or angiotensin-converting enzyme inhibitor users. Additionally, 99 patients (30.9%) had grade 2 or higher RP, and the incidence in the renin-angiotensin system inhibitor group was 17.7% (11 out of 62 patients). Patients in the renin-angiotensin system inhibitors (RASi) group were older and had a higher percentage of males, lower percentage of ECOG score 0, higher percentage of hypertension, and higher percentage of adenocarcinoma than those in the non-RASi group. ECOG score [hazard ratio (HR) = 1.69, *p* = 0.009], history of smoking (HR = 1.76, *p* = 0.049), mean dose (HR = 3.63, *p* = 0.01), and RASi (HR = 0.3, *p* = 0.003) were independent predictive factors for RP. All subgroups benefited from RASi.

**Conclusion:**

This study showed that oral RASi administration has the potential to mitigate the incidence of grade 2 or higher RP in patients with lung cancer undergoing thoracic radiotherapy. To validate and further substantiate these findings, additional prospective research is warranted.

## 1. Introduction

Lung cancer is the leading cause of cancer-related death globally. Locally advanced lung cancer is treated with thoracic radiotherapy (RT), with or without chemotherapy, which can cause radiation-induced injuries, especially radiation pneumonitis (RP), in 5–30% of patients with lung cancer (1). Despite advances in radiation planning and techniques, RP remains a common side effect of thoracic RT. With the expanding indications for thoracic RT in patients with oligometastatic diseases in the era of immunotherapy (2, 3), the population at risk for radiation-induced lung injury is significant. Currently, there is no effective way to prevent or reduce the incidence of RP, except by decreasing the normal lung dosage.

The renin-angiotensin system (RAS) is essential for human bodily fluids, not only in the circulatory system but also in several other tissues and organs. Renin-angiotensin system inhibitors (RASi) are widely used to treat hypertension and related complications. Although prospective randomized clinical trials are lacking to prove their function, several retrospective studies have indicated their protective effects against RP (4–6). A meta-analysis by Sun et al. (7) showed that angiotensin-converting enzyme (ACE) inhibitors (ACEi) decreased the incidence of symptomatic RP in patients with lung cancer.

However, most studies on RASi and RP have been conducted in Caucasians, and there is a lack of studies conducted in Asia. On the other hand, immunotherapy has been used in advanced-stage and metastatic lung cancer (8) and could potentially increase the risk of RP. Therefore, this study aimed to determine the effects of RASi in Chinese patients with lung cancer in the current therapeutic era.

## 2. Patients and methods

### 2.1. Patients and treatment

Patients with lung cancer who underwent thoracic radiation between October 2017 and December 2022 at the Jinan Central Hospital were included in this retrospective study. The inclusion criteria for the study were as follows: (1) a diagnosis of primary lung cancer with pathological diagnosis, including squamous lung cancer, adenocarcinoma, small cell lung cancer, and other pathological types; (2) completion of a standardized staging evaluation before thoracic RT; (3) receiving conventional fractionated RT with a total dose (DT) of ≥45 Gray (Gy), and (4) assessment of RP after RT using computed tomography (CT) scans every 2–3 months for up to 1 year. Data on age, sex, Eastern Cooperative Oncology Group (ECOG) score, smoking history, medical history, histology, clinical stage, and combination therapy were collected. This study was conducted in accordance with the ethical standards of the Declaration of Helsinki and approved by the Research Ethics Committee of the Central Hospital Affiliated to Shandong First Medical University (No. 2022-033-01). The requirement for informed consent was waived by the committee due to the retrospective nature of the study.

### 2.2. Evaluation of the RT plan

Intensity-modulated radiation therapy (IMRT) and volumetric-modulated arc therapy (VMAT) were used for radiation planning and technique. The majority of patients, comprising 84.4% (270 out of 320) were treated with IMRT, and 15.6% (50 out of 320) underwent VMAT. RT plans were retrospectively reviewed by a medical physicist. Data on the mean dose (Dmean) for lung, volume of lung receiving ≥5 Gy (V5), volume of lung receiving ≥10 Gy (V10), volume of lung receiving ≥20 Gy (V20), and volume of lung receiving ≥30 Gy (V30) were collected. The cut-off values of Dmean, V5, V10, V20, and V30 were set at 15 Gy, 60, 50, 28, and 20%, respectively.

### 2.3. RP assessment

RP was diagnosed based on three criteria: (1) pulmonary symptoms such as dyspnea or cough; (2) CT-based imaging changes in the radiated field; and (3) symptoms occurring within 12 months of completing radiation therapy. The Radiation Therapy Oncology Group (RTOG) grading system was used to assess radiation-induced lung toxicity (9). RP staging was performed by two experienced RT physicians, and any inconsistent staging was discussed and resolved.

### 2.4. Statistical analysis

The time to RP was defined as the duration from the end of thoracic radiation to the diagnosis of RP, as confirmed by radiology. The final follow-up was conducted on April 1, 2023. Statistical analyses were performed using SPSS version 24.0 and GraphPad Prism 8.0. The time to RP was estimated using the Kaplan–Meier method and compared using log-rank tests. Cox regression analyses were performed to determine the risk factors. The chi-square test was used to assess the differences in clinical characteristics of patients with RASi. A binary logistic regression was used for subgroup analysis. All tests were two-sided, and a value of *p* less than 0.05 was considered statistically significant.

## 3. Results

### 3.1. Patient characteristics and radiation dose

A total of 320 patients were included in this study. Of these, 245 (76.6%) were male, with a median age of 65 years. The majority of patients (*n* = 282, 88.1%) were diagnosed with non-small cell lung cancer (NSCLC), and 38(11.9%) were diagnosed with small cell lung cancer (SCLC). Totally, 176 (55%) patients had stage III disease, and 103(32.2%) with stage IV disease. 206 (64.3%) patients received concurrent other treatment during radiotherapy, and 195 (60.9%) with chemotherapy-based treatment (Table 1).

**Table 1:** Clinical characteristic of lung cancer patients receiving thoracic radiation.

	Variables	Total *n* = 320 (%)
Age	Mean ± SD	64.9 ± 9.6
	<65	157 (49.1)
	≥65	163 (50.9)
Sex		
	Male	245 (76.6)
	Female	75 (23.4)
ECOG score		
	0	110 (34.3)
	1	169 (52.8)
	2	37 (11.6)
	3	4 (1.3)
History of smoke		
	Yes	197 (61.6)
	No	123 (38.4)
Chronic pulmonary disease		
	Yes	121 (37.8)
	No	198 (61.9)
	Unknown	1 (0.3)
Obstructive pneumonia		
	Yes	149 (46.6)
	No	170 (53.1)
	Unknown	1 (0.3)
Hypertension		
	Yes	119 (37.2)
	No	201 (62.8)
RASi		
	Yes	62 (19.4)
	No	258 (80.6)
Histology		
	Adeno	131 (40.9)
	Squamous	141 (44.1)
	Small cell	38 (11.9)
	Others	10 (3.1)
Stage		
	I–II	41 (12.8)
	III	176 (55.0)
	IV	103 (32.2)
History of surgery		
	Yes	43 (13.4)
	No	277 (86.6)
Combined therapy		
	Chemotherapy alone	143 (44.7)
	Targeted therapy based	45 (14.1)
	Variables	Total *n* = 320 (%)
	Immunotherapy based	19 (5.9)
	None	112 (35.0)
	Unknown	1 (0.3)
Dmean		
	≤15Gy	285 (89.1)
	>15Gy	35 (10.9)
V5		
	≤60%	304 (95.0)
	>60%	16 (5.0)
V10		
	≤50%	316 (98.8)
	>50%	4 (1.3)
V20		
	≤28%	289 (90.3)
	>28%	23 (7.2)
	Unknown	8 (2.5)
V30		
	≤20%	281 (87.8)
	>20%	39 (12.2)

The median DT of the target was 60 Gy, with a mean of 58.4 Gy ± 5.4. The median Dmean was 10.1 Gy with a mean of 9.9 Gy ± 6.5. The median V5 was 42.1%, with a mean of 41.3% ± 12.3. The median V10 was 30.2%, with a mean of 30.3% ± 9.6. The median V20 was 20.0%, with a mean of 19.5% ± 7.0. The median V30 was 14.4%, with a mean of 13.6% ± 5.7.

### 3.2. Difference of characteristics in patients with RASi or not

A total of 62 patients were identified as receiving angiotensin receptor blockers (ARB) or angiotensin-converting enzyme inhibitors (ACEi). Of these, 54 patients were prescribed ARBs (44 were treated with valsartan 80 mg qd, and 10 with telmisartan 40 mg qd) and eight patients were prescribed ACEis (3 captopril 12.5 mg qd, 2 benazepril 5 mg qd, 2 enalapril 5 mg qd, and 1 perindopril 4 mg qd). Additionally, 58 patients had a history of hypertension, the medication was administered by a cardiologist. The drug was administered orally before and during radiotherapy.

Compared with patients who did not take ACEIs or ARBs, patients taking these medications were older, had a higher percentage of males, a lower percentage of ECOG score 0, a higher percentage of hypertension, and a higher percentage of adenocarcinoma. There were no significant differences in Dmean, V5, V10, V20, and V30 between the two groups. A comparison of the patients’ clinical characteristics is presented in Table 2.

**Table 2:** Comparisons of clinical characteristic of patients with RASi or not.

	Variables	non-RASi *n* = 258(%)	RASi *n* = 62(%)	*x* ^2^	*p* value
Age	Mean ± SD	63.6 ± 9.3	70.0 ± 9.1		
	<65	137 (53.1)	20 (32.2)	8.69	0.003
	≥65	121 (46.9)	42 (67.7)		
Sex					
	Male	204 (79.1)	41 (66.1)	4.67	0.031
	Female	54 (20.9)	21 (33.9)		
ECOG score					
	0	97 (37.6)	13 (21.0)	16.04	0.001
	1	135 (52.3)	34 (54.8)		
	2	25 (9.7)	12 (19.4)		
	3	1 (0.4)	3 (4.8)		
History of smoke					
	Yes	163 (63.2)	34 (54.8)	1.67	0.225
	No	95 (36.8)	28 (45.2)		
Chronic pulmonary disease					
	Yes	98 (38.0)	23 (37.1)	0.002	0.968
	No	160 (62.0)	38 (61.3)		
Obstructive pneumonia					
	Yes	119 (46.2)	30 (48.4)	0.19	0.667
	No	139 (53.9)	31 (50.0)		
Hypertension					
	Yes	61 (23.6)	58 (93.5)	104.60	<0.001
	No	197 (76.4)	4 (6.5)		
Histology					
	Adeno	103 (39.9)	28 (45.2)	8.83	0.032
	Squamous	122 (47.3)	19 (30.6)		
	Small cell	25 (9.7)	13 (21.0)		
	Others	8 (3.1)	2 (3.2)		
Stage					
	I–II	30 (11.6)	11 (17.7)	11.87	0.003
	III	154 (59.7)	22 (35.5)		
	IV	74 (28.7)	29 (46.8)		
History of surgery					
	Yes	35 (13.6)	8 (12.9)	0.02	0.891
	No	223 (86.4)	54 (87.1)		
Combined therapy					
	Yes	172 (66.7)	35 (56.5)	2.41	0.121
	No	85 (32.9)	27 (45.2)		
Dmean					
	≤15Gy	229 (88.6)	56 (90.3)	0.125	0.723
	>15Gy	29 (11.2)	6 (9.7)		
V5					
	≤60%	245 (95.0)	59 (95.2)	0.004	0.948
	>60%	13 (5.0)	3 (4.8)		
	Variables	non-RASi *n* = 258(%)	RASi *n* = 62(%)	*x* ^2^	*p* value
V10					
	≤50%	254 (98.4)	62 (100.0)	0.973	0.324
	>50%	4 (1.6)	0 (0)		
V20					
	≤28%	235 (91.1)	54 (87.1)	0.909	0.340
	>28%	23 (8.9)	8 (12.9)		
V30					
	≤20%	228 (88.4)	53 (85.5)	0.390	0.532
	>20%	30 (11.6)	9 (14.5)		

### 3.3. The incidence of RP

The median follow-up period for all patients was 12.5 months (range 0.5–56.7 months). Of the 320 patients included in this study, 147 had no RP, 74 had grade 1 RP, and 99 had ≥ grade 2 RP (30.9%), including 40 with grade 2 RP, 57 with grade 3 RP, and two with grade 4 RP. None of the patients had a grade 5 RP. Nineteen patients were concurrently treated with immunotherapy, and 21.1% (4/19) of them had suffered from ≥ grade 2 RP (2 grade 2 and 2 grade 3).

**Figure 1:**
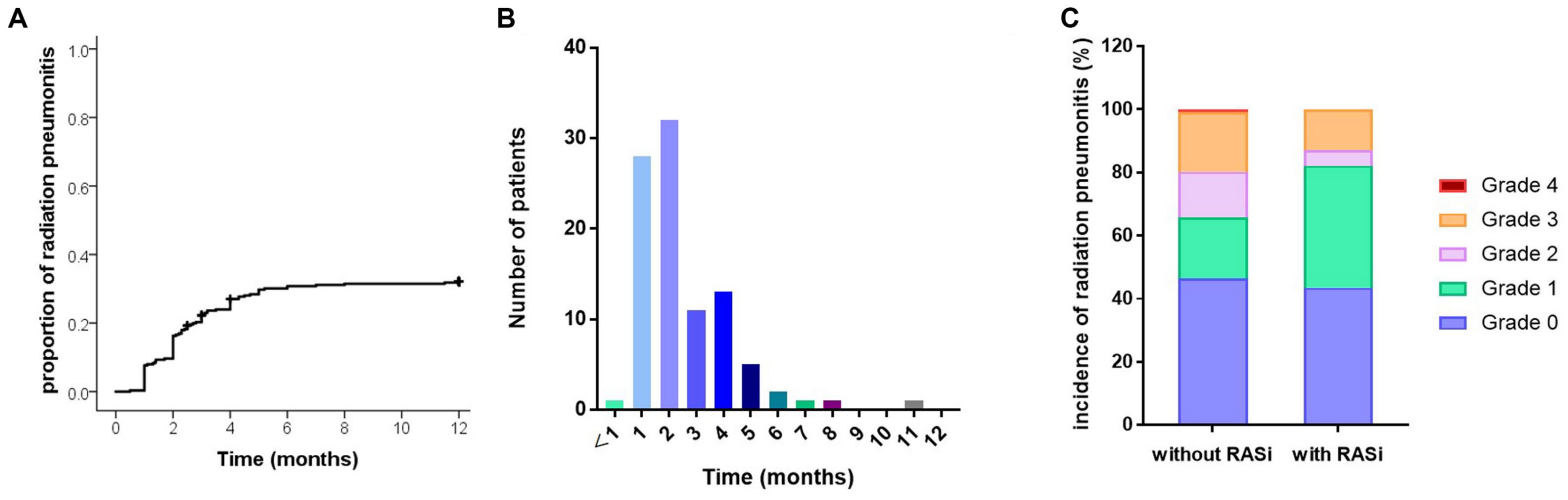
Incidence of radiation pneumonitis (RP). **(A)** Cumulative incidence of grade 2 or above RP. **(B)** Time point of grade 2 or above RP after thoracic radiation. **(C)** Comparison of all grades RP in patients with RASi or not.

The time to ≥ grade 2 RP was collected for 95 patients, and the cumulative occurrence curve is shown in Figure 1A. The median time to ≥ grade 2 RP was 2 months (Figure 1B). A total of 70.5% (67/95) of the cases occurred within 3 months, and 85.1% (81/95) occurred within 4 months. Compared to patients without RASi, those with RASi had a higher incidence of grade 1 RP (38.7% vs. 19.4%, *p* = 0.002), but a lower incidence of grades 2, 3, and 4 RP (4.8% vs. 14.3, 12.9% vs. 19.0, and 0% vs. 0.8%, respectively, *p* = 0.014) (Figure 1C).

### 3.4. Influencing factors of ≥ grade 2 RP

**Figure 2:**
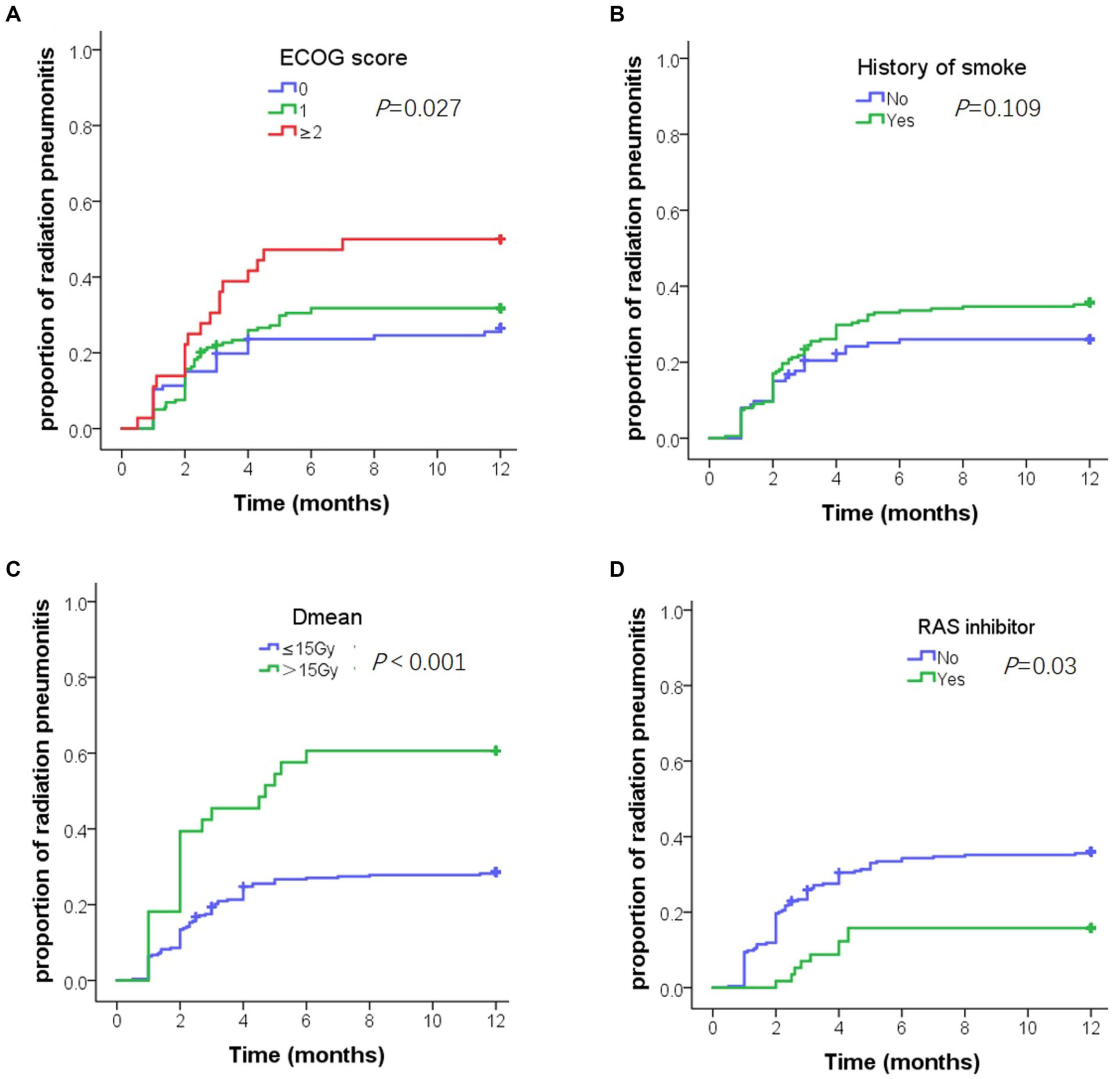
Cumulative incidence of grade 2 or above radiation pneumonitis (RP) in 1 year. **(A)** Cumulative incidence of grade 2 or above RP by Eastern cooperative oncology group score. **(B)** Cumulative incidence of grade 2 or above RP by smoking history. **(C)** Cumulative incidence of grade 2 or above RP by Dmean. **(D)** Cumulative incidence of grade 2 or above RP by renin-angiotensin-system inhibitors.

Univariate analysis showed that the ECOG score, history of smoking, chronic pulmonary disease, Dmean, V5, V20, V30, and RASi were factors influencing ≥ grade 2 RP (Table 3). Multivariate analysis showed that the ECOG score [hazard ratio (HR) = 1.69, *p* = 0.009], history of smoking (HR = 1.76, *p* = 0.049), Dmean (HR = 3.63, *p* = 0.01), and RASi (HR = 0.3, *p* = 0.003) were the independent predictive factors (Table 4). Figure 2 shows the time to ≥ grade 2 RP curve in patients with risk factors, who had a higher and earlier ≥ grade 2 RP.

**Table 3:** Univariate analysis of the influencing factors of ≥2 grade RP in patients with thoracic radiation.

	Variables	≥2 RP rate (%)	OR	95%CI	*p* value
Age					
	≥65	35.6	1.56	0.97–2.52	0.068
	<65	26.1			
Sex					
	Male	33.5	0.58	0.32–1.06	0.079
	Female	22.7			
ECOG score					
	0	25.5	1.50	1.06–2.12	0.022
	1	30.8			
	2	45.9			
	3	50.0			
History of smoke					
	Yes	35.5	1.79	1.07–2.97	0.025
	No	23.6			
Chronic pulmonary disease					
	Yes	39.7	1.95	1.20–3.16	0.007
	No	25.3			
Obstructive pneumonia					
	Yes	31.5	1.08	0.67–1.73	0.766
	No	30.0			
Hypertension					
	Yes	31.8	0.89	0.55–1.46	0.650
	No	29.4			
Histology					
	Adeno	26.4	1.17	0.87–1.58	0.305
	Squamous	37.6			
	Small cell	31.6			
	Others	20.0			
Stage					
	I–II	24.4	0.99	0.86–1.28	0.941
	III	36.3			
	IV	25.2			
History of surgery					
	Yes	27.9	0.85	0.41–1.73	0.644
	No	31.4			
Combined therapy					
	Yes	33.3	1.43	0.86–2.39	0.170
	No	25.9			
Dmean					
	>15Gy	60.0	3.98	1.93–8.21	<0.001
	≤15Gy	27.3			
V5					
	>60%	62.5	4.03	1.42–11.41	0.009
	Variables	≥2 RP rate (%)	OR	95%CI	*p* value
	≤60%	29.3			
V10					
	>50%	75.0	6.88	0.71–66.94	0.097
	≤50%	30.4			
V20					
	>28%	48.4	2.29	1.08–4.84	0.030
	≤28%	29.1			
V30					
	>20%	48.7	2.39	1.21–4.71	0.012
	≤20%	28.5			
RASi					
	Yes	17.7	0.42	0.21–0.84	0.014
	No	34.1			

**Table 4:** Multivariate analysis of the influencing factors of ≥2 grade RP in patients with thoracic radiation.

Variables	OR	95%CI	*p* value
ECOG score	1.69	1.14–2.51	0.009
History of smoke (Yes vs. No)	1.76	1.00–3.09	0.049
Chronic pulmonary disease (Yes vs. No)	1.55	0.91–2.64	0.107
Dmean (>15Gy vs. ≤15Gy)	3.63	1.36–9.69	0.010
V5 (>60% vs. ≤60%)	2.26	0.66–7.74	0.193
V20 (>28% vs. ≤28%)	0.74	0.21–2.61	0.642
V30 (>20% vs. ≤20%)	1.52	0.52–4.47	0.444
RASi (Yes vs. No)	0.30	0.13–0.66	0.003

### 3.5. The benefit of ACEi or ARB in subgroup analysis

**Figure 3:**
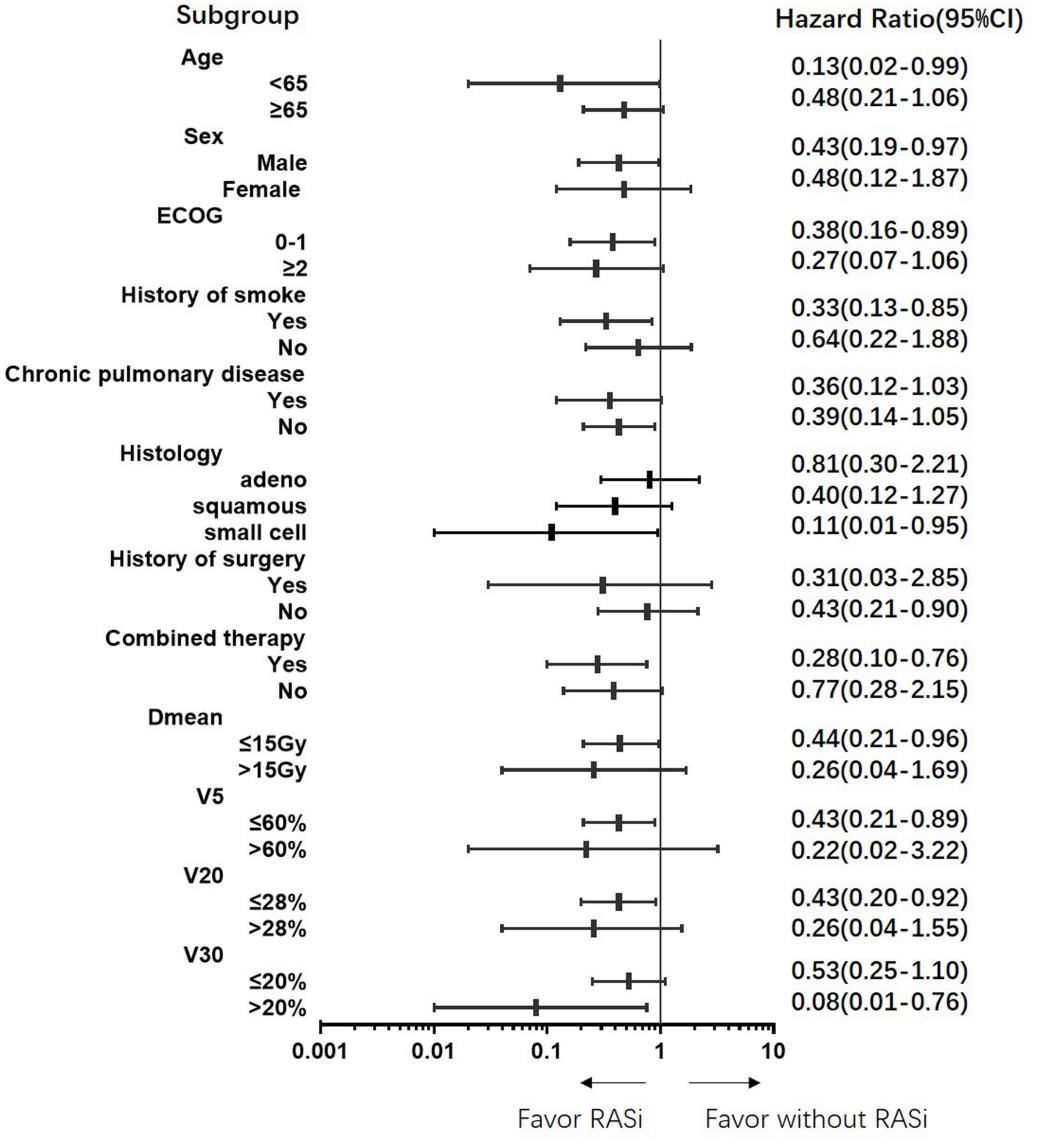
Protective benefit of renin-angiotensin-system inhibitors on grade 2 or above radiation pneumonitis in all subgroups.

All subgroups benefited from ACEi or ARB treatment (Figure 3). Specifically, those with a higher risk of RP, such as those with a smoking history and higher Dmean, V5, V20, and V30, benefited more from ACEi or ARB.

## 4. Discussion

RT is a common curative treatment for patients with lung cancer; however, pneumonitis and fibrosis can cause severe complications. Corticosteroids are currently the main treatment for acute pneumonitis (10, 11), making it crucial to identify protective measures. In our study of Chinese patients, we found that RASi significantly reduced the risk of grade 2 RP, regardless of concurrent chemotherapy/immunotherapy.

We observed a higher rate of pneumonitis (30.9%) in our study population compared to previous reports. This may be due to the fact that our patients included had higher ECOG scores, smoking history, and received radical doses. The occurrence of RP is related to the physical parameters of radiotherapy and systemic therapy, including synchronous chemotherapy, immunotherapy, and targeted therapy. In addition, the nutritional status of patients and the associated basic diseases also impact the generation of RP (12). In our study, ECOG score, history of smoking, mean dose, and RASi were independent predictive factors for RP. The ECOG score is a key predictive and prognostic factor for many anticancer therapies (13), and our data showed that patients with higher scores had a higher risk of RP. Additionally, smoking history was a significant risk factor for RP, which is consistent with the results of previous studies (1). Dmean was the strongest predictor of RP (HR = 3.63) and was found to be a radiation dose-dependent toxicity. Identifying effective protective measures against RP is critical to improving patient outcomes. The RASi was found to be a protective factor against grades 2, 3, and 4 RP. Our findings suggest that RASi may be a viable option for reducing the risk of RP, particularly for patients at higher risk due to factors such as smoking history and higher radiation doses.

In this study, we included 103 patients with stage IV, of whom 25.2% developed RP. We did not observe any improvement in the incidence of RP. Most patients with metastasis had received systemic therapy for disease control. The oligometastatic hypothesis suggests that some patients with a limited burden of metastasis can be cured with local therapy (14). Local radiation therapy has been shown to prolong survival in patients with metastatic lung cancer after effective systemic therapy (15). RP has a relatively high incidence in patients who receive RT combined with systemic treatments, especially immunotherapy (16–18). Although the combination therapy may have had an effect on RP in our study (HR = 1.43), the effect was not statistically significant. Moreover, a real-world study showed that immunotherapy within 90 days of RT was not associated with an increased risk of serious adverse events (19). Another study showed that the adoption of immunotherapy consolidation was associated with an increase in grade ≥ 2 pneumonitis but not grade ≥ 3 pneumonitis in patients with locally advanced NSCLC receiving concurrent chemoradiation therapy (20). Therefore, it appears safe to administer immunotherapy without interruption during thoracic RT in selected patients with stricter lung dose constraints.

We analyzed the clinical characteristics of the patients treated with RASi. In our real-world study, 93.5% of the patients treated with RASi had a history of hypertension and were taking drugs to control blood pressure. Patients in the RASi group were older and had worse ECOG scores and higher disease stages; however, other clinical characteristics were balanced between the RASi and non-RASi groups. Nonetheless, patients in the RASi group had less grade 2 and grade 3 RP than those without RASi, and some ≥ grade 2 patients were able to decrease to grade 1. Subgroup analysis showed that RASi could decrease RP risk in all subgroups, particularly in those with V30 > 20%. Therefore, RASi may be a promising adjunct to thoracic RT (21). Additionally, studies also showed ACEi/ARB associated with improved survival outcomes in cancer patients receiving anti-vascular endothelial growth factor therapy and immune checkpoint inhibitors therapy (22, 23).

The potential protective mechanisms of RASi against RP have recently been elucidated (24, 25). The pathogenesis of radiation-induced lung toxicity is a complex, multi-step process involving several residents and recruited immune cells in the lungs. It is initiated and perpetuated via pleiotropic inter-and intracellular communication and signaling events (16, 26). Ang II is one of the important molecules involved in this process. The AngII/AT1R axis contributes to tissue damage and remodeling by upregulating the profibrogenic and proinflammatory pathways and ROS production via the activation of NADPH oxidase. ACEs can convert angiotensin I (Ang I) to active angiotensin II (Ang II). Therefore, inhibiting the AngII/AT1R signaling pathway is a potential strategy for preventing or reducing radiation-induced injury (27). In addition, recent research has also shown that radiation-induced ACE activation within the immune compartment promotes the pathogenesis of radiation pneumonitis, whereas ACEis can suppress the activation of proinflammatory immune cell subsets (28, 29). Among the patients in the RASi group, 87.1% were treated with ARB. Both ACEi and ARB act on the RAS. ARBs are angiotensin II receptor antagonists, which are highly selective for AT1. Although there is no significant difference between the effectiveness of the ARBs and ACEi as first-line treatment for hypertension, ARBs have a better safety profile (30). Patients taking ACEi have significantly higher risks of angioedema, cough, pancreatitis, and gastrointestinal bleeding than those taking ARBs (31).

Our study had several limitations. Firstly, this was a retrospective study, and the influencing factors were complicated, which need to be confirmed by further prospective studies. Although two prospective studies have been conducted on the function of ACEi in the RP (32, 33), they were discontinued due to slow enrollment and did not reach the intended endpoint. One reason for the low enrollment rate in RTOG 0123 was that most patients did not receive drug intervention with captopril. Further prospective clinical trials of ARBs may improve their tolerance. Our center is currently enrolling patients in a prospective clinical trial of ARBs (ChiCTR2200062762). Secondly, the combination therapies in this study were diverse, including chemotherapy, immunotherapy, and targeted therapies, such as endostatin, bevacizumab, and small-molecule tyrosine kinase inhibitors. Due to the limited sample size, no further stratified analyses were performed. Thirdly, the median follow-up period of this study was 12.5 months to analyze the incidence of RP. However, prognostic and survival information was not collected.

Our study demonstrated that oral RAS inhibitors have a protective effect during thoracic RT in patients with stage III and IV lung cancers. They can reduce the incidence of grade 2 or higher radiation pneumonitis and improve the occurrence.

## Data availability statement

The original contributions presented in the study are included in the article/supplementary material, further inquiries can be directed to the corresponding author.

## Ethics statement

The studies involving humans were approved by This study was conducted in accordance with the ethical standards of the Declaration of Helsinki and approved by the Research Ethics Committee of the Central Hospital Affiliated to Shandong First Medical University (No. 2022-033-01). The studies were conducted in accordance with the local legislation and institutional requirements. The ethics committee/institutional review board waived the requirement of written informed consent for participation from the participants or the participants’ legal guardians/next of kin because The requirement for informed consent was waived by the committee due to the retrospective nature of the study.

## Author contributions

YZ: Data curation, Writing – original draft. CC: Data curation, Writing – original draft. ZW: Data curation, Writing – original draft. YL: Supervision, Writing – review & editing. MZ: Writing – review & editing. HZ: Writing – review & editing. CS: Supervision, Writing – review & editing. MS: Conceptualization, Writing – review & editing.
